# Data on yields, sugars and glycosidic-linkage analyses of coffee arabinogalactan and galactomannan mixtures and optimization of their microwave assisted extraction from spent coffee grounds

**DOI:** 10.1016/j.dib.2019.103931

**Published:** 2019-04-19

**Authors:** Cláudia P. Passos, Alisa Rudnitskaya, José M.M.G.C. Neves, Guido R. Lopes, Manuel A. Coimbra

**Affiliations:** a QOPNA & LAQV-REQUIMTE, Department of Chemistry, University of Aveiro, 3810-193 Aveiro, Portugal; b CESAM, Department of Chemistry, University of Aveiro, 3810-193 Aveiro, Portugal

**Keywords:** Coffee residue, Arabinogalactans, Galactomannans, Polysaccharides, Methylation analysis, Microwave assisted extraction

## Abstract

The data presented here are related to the research paper entitled “Structural features of spent coffee grounds water-soluble polysaccharides: towards tailor-made microwave assisted extractions” [1]. Microwave assisted extraction conditions were applied to spent coffee grounds for recovery of polysaccharides, namely arabinogalactans and galactomannans. Following an experimental design testing temperature, time, and alkali conditions as influence factors during microwave assisted extraction, this article reports the response data for the total extracted mass, sugars yield (including arabinogalactans and galactomannans total content, and mass ratio), and structural features (including degree of polymerization and degree of branching) for each set of operating conditions. In addition, it provides gas chromatography–mass spectrometry (GC–MS) chromatograms (and respective GC–MS spectra) of arabinogalactan and galactomannan mixtures with different structural features corresponding to representative microwave treatment conditions.

**Table:** Specifications table

Subject area	*Chemistry*
More specific subject area	*Sugars and linkage analyses of coffee arabinogalactan and galactomannan mixtures; chromatographic examples for the experimental design obtained during the optimization of polysaccharides microwave assisted extractions*
Type of data	*Tables and figures*
How data was acquired	*Microwave assisted extraction of polysaccharides from spent coffee grounds was conducted using a microwave Labstation (MicroSYNTH, Milestone srl.)* *Sugar contents were determined as alditol acetates by GC (GC-FID, Perkin-Elmer)* *Glycosidic-linkage analyses were carried out using partially methylated alditol acetates by GC-MS (Agilent Technologies 6890 N Network with a 5973 Mass selective detector)* *Statistical analyses was done using Matlab 9.5 (R2018b)*
Data format	*Raw and analyzed*
Experimental factors	*Microwave assisted treatment of spent coffee grounds at different temperatures, times and application of alkali using a full factorial design*
Experimental features	Sugars and linkage analyses of coffee arabinogalactan and galactomannan mixtures with chromatographic examples for the experimental design obtained during the optimization of polysaccharides microwave assisted extractions from spent coffee grounds for tailor-made applications
Data source location	*Spent coffee grounds were obtained from a commercial lot of Delta Cafés Platina (Portugal) after beverage preparation in a local cafeteria*
Data accessibility	*Data is provided with this article*
Related research article	C.P. Passos, A. Rudnitskaya, J.M.M.G.C. Neves, G.R. Lopes, D.V. Evtuguin, M.A. Coimbra, Structural features of spent coffee grounds water-soluble polysaccharides: towards tailor-made microwave assisted extractions, Carbohydr. Polym. 214, 2019, 53–61 [1].

**Table:** 

**Value of the data** • Mass spectrometry data can be used to identify galactomannans and/or arabinogalactans from different sources. • Data about total soluble solids mass yield [η_total soluble solids_, (%, w/w)]; total sugars yield (η_sugars_, %); arabinogalactans (AG) sugar content [η_AG_, (mg_AG_/g_SCG_)] and (η_AG_, %); galactomannans (GM) sugar content [η_GM_, (mg_GM_/g_SCG_)] and (η_AG_, %); degree of polymerization (DP); and degree of branching (DB), used for defining the specific microwave operating conditions for carbohydrates extraction, are provided. • The detailed data on the chemical and structural characterization of spent coffee grounds microwave assisted extracted samples can be used for comparative purposes with galactomannans or arabinogalactans of other sources/conditions of extraction. • Contour plot representation of the interaction between the operating conditions versus the data obtained are explained, allowing to define the areas of similar response for the different operating conditions. • Different contour plots can be made with the presented data towards the definition of the operating conditions required for specific extraction of polysaccharide characteristics.

## 1 Data

**Fig. 1:**
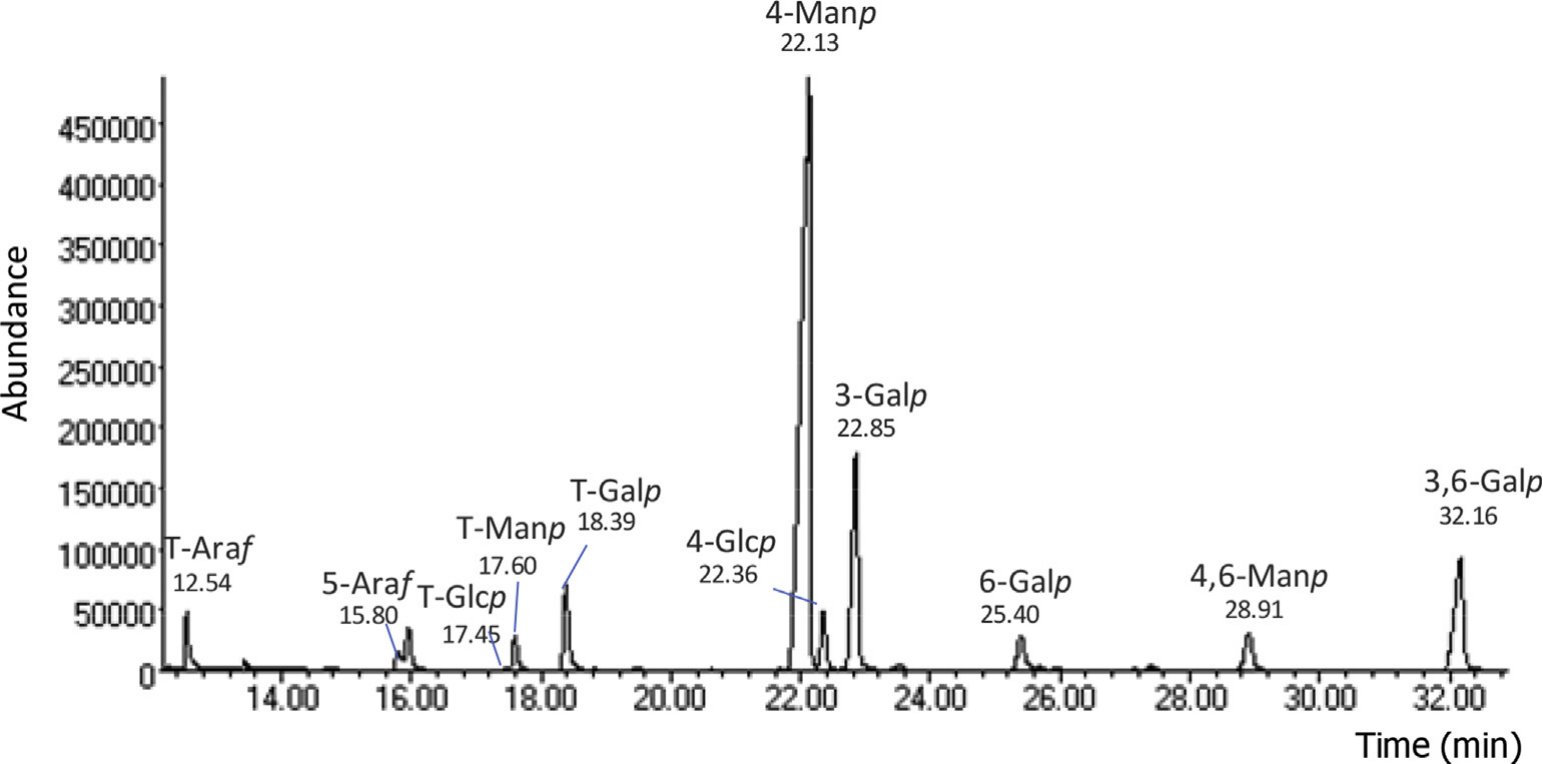
Chromatogram of a sample B at 140 °C, 2 min under NaOH (respective data are shown in Table 1).

**Fig. 2:**
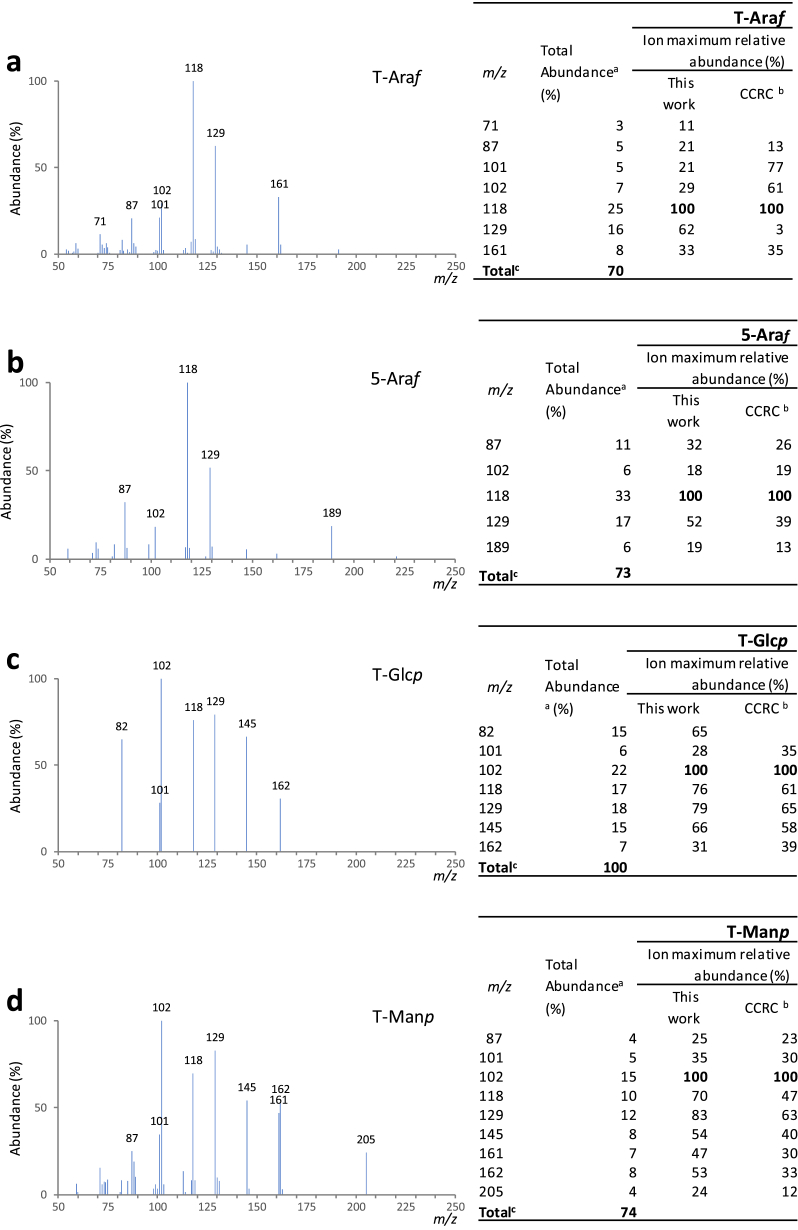

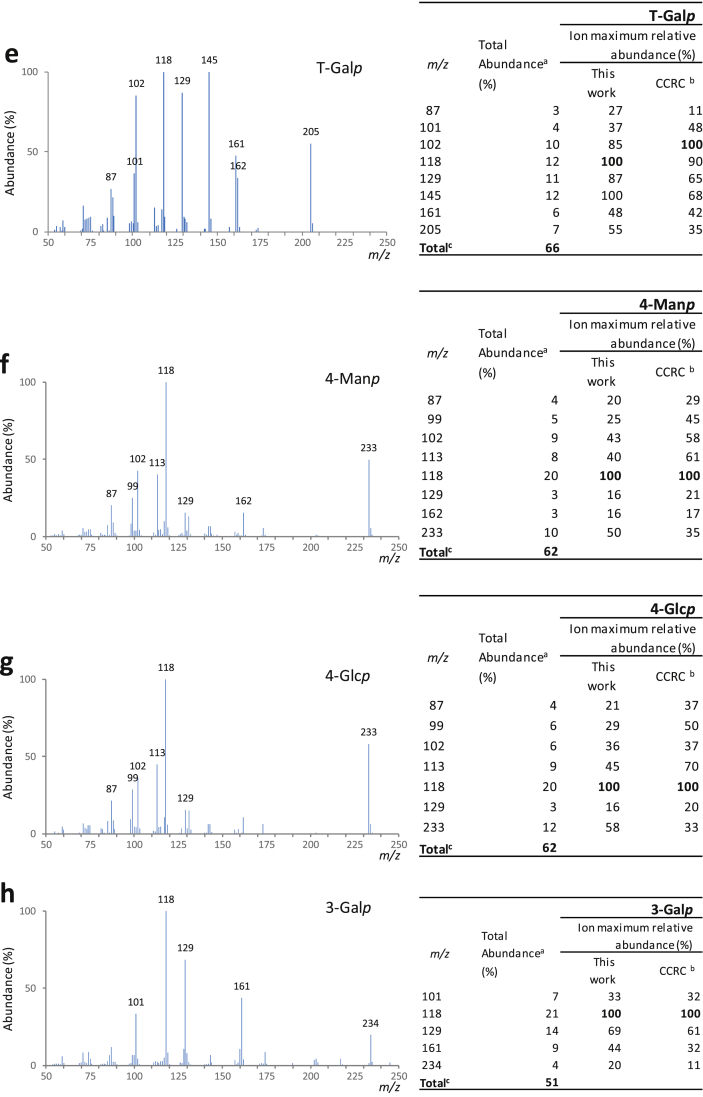

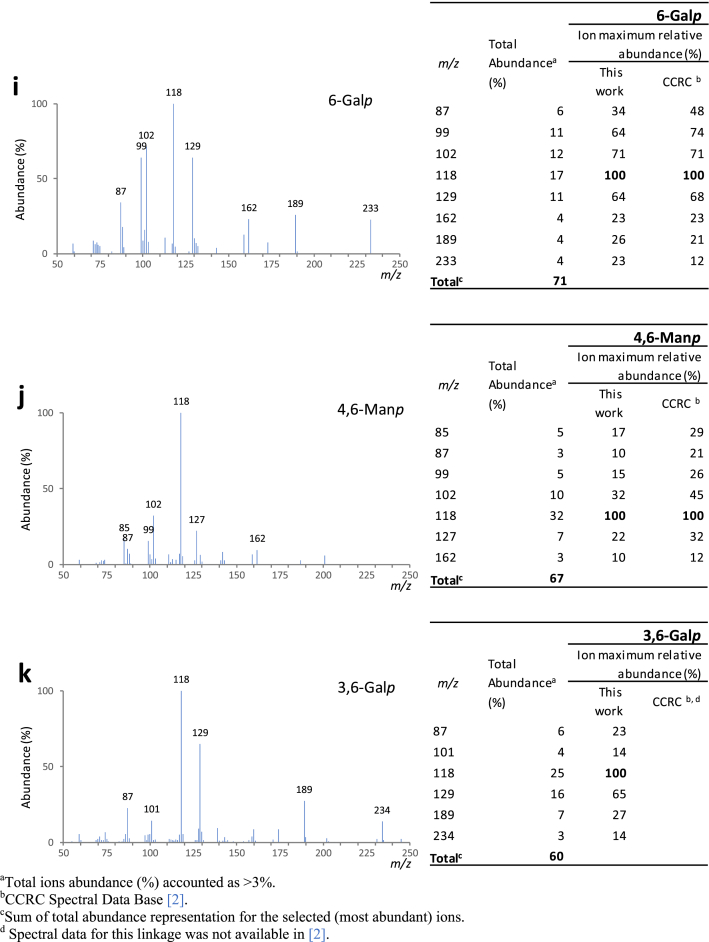
Mass spectra for each one of the major partially methylated alditol acetates identified in the chromatogram represented in Fig. 1 (sample B at 140 °C, 2 min under NaOH). Also represented are the total abundance (%) and the ion maximum relative abundances: a) T-Ara*f*; b) 5-Ara*f*; c) T-Glc*p*; d) T-Man*p*; e) T-Gal*p*; f) 4-Man*p*; g) 4-Glc*p*; h) 3-Gal*p*; i) 6-Gal*p*; j) 4,6-Man*p*; k) 3,6-Gal*p*.

The data presented in Section 1.1 include gas chromatography-mass spectrometry (GC–MS) chromatogram for a mixture of galactomannans (GM) and arabinogalactans (AG) (Fig. 1) and respective GC–MS spectra (Fig. 2). The data include also the total abundance (%) and the ion maximum relative abundances (%) and the comparison with the partially methylated alditol acetates (PMAA) spectra of a spectral database (CCRC) [2].

**Fig. 3:**
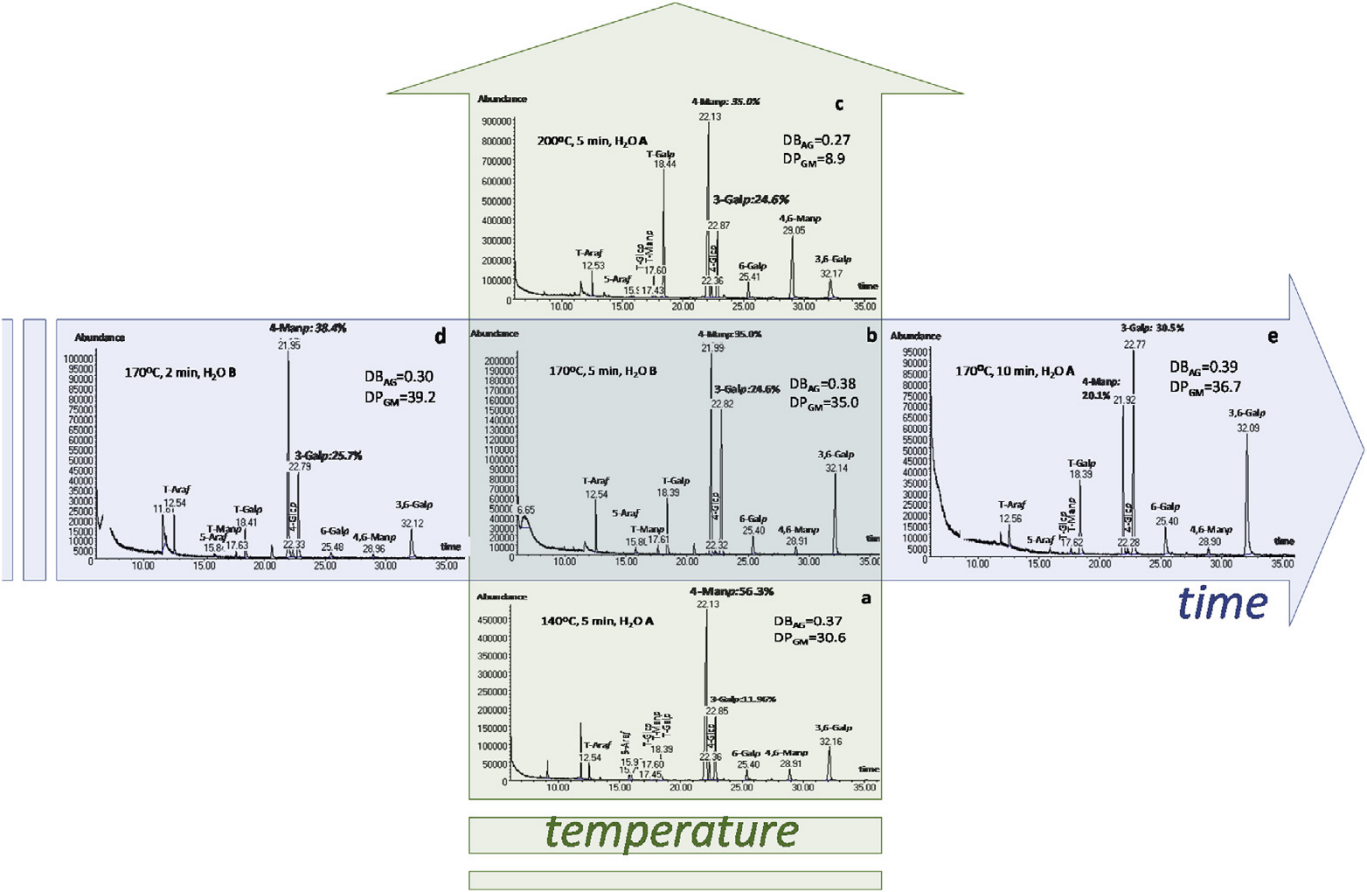
Chromatograms Map for glycosidic-linkage analyses for different microwave assisted operating conditions. Chromatograms obtained towards increasing temperature (vertical): a) 140 °C; b) 170 °C; c) 200 °C. Chromatograms obtained with increasing time (horizontal): d) 2 min; b) 5 min; e) 10 min.

In Section 1.2, the data for sugar and glycosidic-linkage analysis after each one of the microwave assisted treatments are presented. The effect of temperature is illustrated by the chromatograms (Fig. 3) of the extracts obtained at 140 °C (Fig. 3a), 170 °C (Fig. 3b), and 200 °C (Fig. 3c). The time effect was also followed at 2 min (Fig. 3d), 5 min (Fig. 3b), and 10 min (Fig. 3e). Detailed information on glycosidic-linkage (M) and sugars composition (A) of the samples digested at 140 °C, 170 °C, and 200 °C is presented in Table 1, Table 2, Table 3, respectively.

**Table 1:** Chemical characterization of water-soluble material obtained under microwave assisted conditions using aqueous/or dilute alkali treatments at 140 °C. The data includes total soluble solids mass yield [*η*_total soluble solids_, (%, w/w)]; total sugars yield (*η*_sugars_, %); arabinogalactans (AG) sugar content [*η*_AG_, (mg_AG_/g_SCG_)] and (*η*_AG_, %); galactomannans (GM) sugar content [*η*_GM_, (mg_GM_/g_SCG_)] and (*η*_GM_, %); degree of polymerization (DP); and degree of branching (DB).

t (min)		Aqueous						NaOH					
		2 min		5 min		10 min		2 min		5 min		10 min	
		A	B	A	B	A	B	A	B	A	B	A	B
*η*_total soluble solids_ (%)		9.0	10.3	8.7	8.3	13.8	8.9	8.4	8.5	7.3	7.9	8.5	8.9
*η*_sugars_ (%)		40.0	43.5	64.7	42.0	47.6	54.7	36.4	28.1	36.0	34.0	43.6	41.8
Linkage (%)													
T-Ara*f*		2.6	3.8	4.5	2.4	3.7	3.2	1.8	2.6	3.0	5.0	2.9	3.3
5-Ara*f*		0.0	0.8	1.7	0.9	1.9	1.2	1.0	1.5	1.1	0.6	1.2	0.9
Total Ara (M)		**2.6**	**4.6**	**6.2**	**3.3**	**5.6**	**4.4**	**2.8**	**4.1**	**4.0**	**5.6**	**4.1**	**4.2**
(A)		**(10.9)**	**(11.1)**	**(11.2)**	**(11.0)**	**(11.1)**	**(10.5)**	**(10.7)**	**(8.7)**	**(9.1)**	**(10.1)**	**(9.2)**	**(10.7)**
T-Man*p*		1.2	1.4	2.0	1.6	2.1	1.5	1.5	2.2	1.1	1.2	1.7	1.4
4-Man*p*		69.5	64.6	56.3	59.6	50.9	53.2	52.5	52.3	38.4	48.7	46.9	41.6
4,6-Man*p*		1.7	1.6	3.3	2.5	3.2	2.2	2.7	4.3	1.8	1.2	2.3	2.1
Total Man (M)		**72.4**	**67.5**	**61.6**	**63.7**	**56.3**	**56.9**	**56.7**	**58.8**	**41.3**	**51.0**	**50.8**	**45.1**
(A)		**(46.5)**	**(47.0)**	**(47.7)**	**(47.3)**	**(42.8)**	**(43.8)**	**(46.2)**	**(46.7)**	**(42.1)**	**(45.0)**	**(43.1)**	**(44.1)**
T-Gal*p*		3.7	4.3	6.0	4.7	6.8	4.7	12.8	4.7	8.1	6.5	6.1	4.9
6-Gal*p*		1.6	1.4	2.9	2.3	3.6	2.8	2.3	4.1	3.8	1.9	3.3	2.7
3-Gal*p*		12.4	12.9	11.9	14.4	15.3	18.9	12.8	12.7	25.7	20.6	18.1	19.1
3,6-Gal*p*		6.1	8.3	10.3	10.9	10.8	11.4	9.6	12.2	15.6	12.8	12.8	13.3
Total Gal (M)		**23.6**	**26.9**	**31.1**	**32.3**	**36.6**	**37.8**	**37.6**	**33.7**	**53.1**	**41.7**	**40.4**	**40.0**
(A)		**(37.1)**	**(37.3)**	**(36.7)**	**(37.1)**	**(40.8)**	**(41.3)**	**(37.0)**	**(38.5)**	**(44.7)**	**(39.7)**	**(42.9)**	**(40.7)**
T-Glc*p*		0.0	0.0	0.1	0.0	0.1	0.1	0.1	0.3	0.1	0.0	1.0	0.0
4-Glc*p*		1.3	1.0	1.1	0.7	1.4	0.9	2.9	2.8	1.5	1.6	3.6	10.8
Total Glc (M)		**1.3**	**1.0**	**1.2**	**0.7**	**1.6**	**0.9**	**3.0**	**3.1**	**1.6**	**1.6**	**4.7**	**10.8**
(A)		**(3.8)**	**(3.0)**	**(2.9)**	**(3.1)**	**(3.6)**	**(2.9)**	**(4.5)**	**(4.7)**	**(2.5)**	**(3.5)**	**(3.3)**	**(3.1)**
**AG**	***η***_**AG**_ (mg_AG_/g_SCG_)	7.8	13.4	19.0	11.6	25.5	19.4	11.6	8.0	14.5	12.3	15.6	15.5
	***η***_**AG**_ (%)^a^	25	30	34	33	39	40	38	34	55	46	42	42
	DP_AG_	6.0	5.9	4.6	6.4	4.9	7.6	2.7	6.2	6.4	6.3	6.2	7.7
	DB_AG_	0.28	0.33	0.37	0.37	0.33	0.32	0.28	0.42	0.30	0.32	0.34	0.35
**GM**	***η***_**GM**_ (mg_GM_/g_SCG_)	23.7	30.9	36.3	23.1	39.0	28.7	18.2	15.0	11.3	14.0	19.6	17.4
	***η***_**GM**_ (%)^b^	74	69	65	66	60	59	59	63	43	52	53	47
	DP_GM_	62.3	48.8	30.6	39.5	26.3	38.2	37.9	26.6	39.2	43.3	30.8	32.1
	DB_GM_	0.02	0.02	0.05	0.04	0.06	0.04	0.05	0.07	0.04	0.02	0.05	0.05
AG/GM		0.3	0.4	0.5	0.5	0.7	0.7	0.6	0.5	1.3	0.9	0.8	0.9

Reprint from Passos et al., Ref. [1]. Samples A and B are the duplicate samples respectively obtained at reactor A and B in each microwave run. (M) Glycosidic-linkage composition of polysaccharides was determined as partially methylated alditol acetated by methylation analysis with GC-MS. (A) Sugar composition determined by derivatization to alditol acetates and analysis by GC-FID.

a [AG/(AG + GM)].

b [GM/(AG + GM)]. DP – Degree of polymerization. DB – Degree of Branching.

**Table 2:** Chemical characterization of water-soluble material obtained under microwave assisted conditions using aqueous/or dilute alkali treatments at 170 °C. The data includes total soluble solids mass yield [*η*_total soluble solids_, (%, w/w)]; total sugars yield (*η*_sugars_, %); arabinogalactans (AG) sugar content [*η*_AG_, (mg_AG_/g_SCG_)] and (*η*_AG_, %); galactomannans (GM) sugar content [*η*_GM_, (mg_GM_/g_SCG_)] and (*η*_GM_, %); degree of polymerization (DP); and degree of branching (DB).

t (min)		Aqueous						NaOH					
		2 min		5 min		10 min		2 min		5 min		10 min	
		A	B	A	B	A	B	A	B	A	B	A	B
*η*_total soluble solids_ (%)		12.9	11.3	13.4	16.9	20.2	16.9	12.6	9.9	14.6	17.9	23.9	17.8
*η*_sugars_ (%)		58.3	55.8	65.1	58.3	71.6	65.0	58.8	56.8	62.9	60.6	66.9	62.8
Linkage (%)													
T-Ara*f*		3.2	3.0	3.4	3.9	2.0	2.6	4.5	5.1	4.0	5.1	2.6	2.4
5-Ara*f*		1.6	1.1	1.1	1.1	0.0	0.5	0.9	0.6	0.8	0.4	0.5	0.3
Total Ara (M)		**4.8**	**4.0**	**4.5**	**5.0**	**2.0**	**3.1**	**5.4**	**5.7**	**4.8**	**5.5**	**3.1**	**2.6**
(A)		**(11.0)**	**(11.1)**	**(11.6)**	**(11.8)**	**(6.9)**	**(10.5)**	**(11.8)**	**(12.0)**	**(12.0)**	**(10.7)**	**(7.5)**	**(7.5)**
T-Man*p*		2.1	1.1	1.4	1.1	0.6	1.6	1.0	0.8	1.2	0.9	2.5	1.3
4-Man*p*		40.3	38.4	37.8	35.0	20.1	26.0	30.6	36.3	26.3	24.3	24.0	22.5
4,6-Man*p*		4.1	1.8	3.1	1.8	1.3	0.9	1.5	0.6	2.4	0.7	2.0	0.8
Total Man (M)		**46.5**	**41.3**	**42.3**	**37.9**	**22.0**	**28.5**	**33.2**	**36.9**	**29.9**	**25.8**	**28.5**	**24.5**
(A)		**(36.2)**	**(35.5)**	**(32.3)**	**(32.0)**	**(30.6)**	**(26.8)**	**(30.1)**	**(29.9)**	**(27.3)**	**(25.7)**	**(28.4)**	**(29.2)**
T-Gal*p*		8.0	8.1	8.0	7.8	10.5	10.6	9.3	7.6	9.4	10.2	13.4	11.6
6-Gal*p*		6.1	3.8	5.5	3.6	5.6	4.8	4.7	1.2	5.8	3.0	7.8	4.8
3-Gal*p*		16.4	25.7	20.0	24.6	30.5	33.1	25.7	32.8	26.5	34.8	27.1	34.9
3,6-Gal*p*		14.1	15.6	18.7	20.7	28.8	19.5	21.1	15.2	22.1	19.9	19.1	17.7
Total Gal (M)		**44.7**	**53.1**	**52.2**	**56.7**	**75.4**	**68.0**	**60.9**	**56.8**	**63.7**	**67.9**	**67.4**	**68.9**
(A)		**(49.6)**	**(50.1)**	**(53.3)**	**(53.5)**	**(60.3)**	**(60.0)**	**(55.3)**	**(55.0)**	**(58.0)**	**(60.8)**	**(62.0)**	**(61.3)**
T-Glc*p*		0.2	0.1	0.0	0.0	0.0	0.0	0.0	0.0	0.1	0.0	0.0	0.0
1,4-Glc*p*		3.1	1.5	1.0	0.5	0.6	0.5	0.6	0.7	1.4	0.8	1.0	3.9
Total Glc (M)		**3.3**	**1.6**	**1.0**	**0.5**	**0.6**	**0.5**	**0.6**	**0.7**	**1.6**	**0.8**	**1.0**	**3.9**
(A)		**(2.2)**	**(2.3)**	**(1.8)**	**(1.7)**	**(1.7)**	**(1.9)**	**(2.0)**	**(2.1)**	**(1.7)**	**(2.1)**	**(1.6)**	**(1.6)**
**AG**	***η***_**AG**_ (mg_AG_/g_SCG_)	34.6	35.0	46.6	58.7	110.4	77.3	47.9	34.7	60.9	79.0	109.4	78.9
	***η***_**AG**_ (%)^a^	46	55	54	60	76	70	65	62	66	73	68	71
	DP_AG_	5.1	6.4	6.2	7.0	7.1	6.4	6.4	7.4	6.6	6.6	4.9	5.9
	DB_AG_	0.35	0.30	0.38	0.38	0.39	0.29	0.36	0.27	0.36	0.30	0.29	0.26
**GM**	***η***_**GM**_ (mg_GM_/g_SCG_)	38.1	27.2	39.5	38.9	33.7	32.3	25.6	21.0	29.7	28.8	48.7	28.3
	***η***_**GM**_ (%)^b^	51	43	45	40	23	29	35	37	32	26	30	25
	DP_GM_	22.4	39.2	31.2	35.0	36.7	17.5	31.7	47.1	24.6	29.5	11.5	19.6
	DB_GM_	0.09	0.04	0.07	0.05	0.06	0.03	0.05	0.02	0.08	0.03	0.07	0.03
AG/GM		1.3	1.2	1.5	3.3	2.4	1.9	1.7	2.1	2.7	2.2	2.8	

Samples A and B are the duplicate samples respectively obtained at reactor A and B in each microwave run. (M) Glycosidic-linkage composition of polysaccharides was determined as partially methylated alditol acetated by methylation analysis with GC-MS. (A) Sugar composition determined by derivatization to alditol acetates and analysis by GC-FID.

a [AG/(AG + GM)].

b [GM/(AG + GM)]. DP – Degree of polymerization. DB – Degree of Branching.

**Table 3:** Chemical characterization of water-soluble material obtained under microwave assisted conditions using aqueous/or dilute alkali treatments at 200 °C. The data includes total soluble solids mass yield [*η*_total soluble solids_, (%, w/w)]; total sugars yield (*η*_sugars_, %); arabinogalactans (AG) sugar content [*η*_AG_, (mg_AG_/g_SCG_)] and (*η*_AG_, %); galactomannans (GM) sugar content [*η*_GM_, (mg_GM_/g_SCG_)] and (*η*_GM_, %); degree of polymerization (DP); and degree of branching (DB).

t (min)		Aqueous				NaOH						
		2 min		5 min		2 min			5 min			
		A	B	A	B	A	B		A		B	
*η*_total soluble solids_ (%)		22.4	19.2	27.2	26.8	17.8	15.4		22.1		21.3	
*η*_sugars_ (%)		45.5	50.1	52.3	49.6	58.1	59.4		68.0		42.8	
Linkage (%)												
T-Ara*f*		3.7	3.1	2.6	1.9	3.8		3.2		2.7		2.3
5-Ara*f*		1.1	0.0	0.0	0.0	0.9		0.3		0.8		0.5
Total Ara (M)		**5.0**	**3.1**	**2.6**	**1.9**	**4.7**		**3.5**		**3.6**		**2.8**
(A)		**(6.5)**	**(6.7)**	**(4.5)**	**(2.8)**	**(10.7)**		**(7.6)**		**(7.9)**		**(6.2)**
T-Man*p*		4.1	4.2	3.5	1.8	1.8		1.9		3.7		3.4
4-Man*p*		22.7	20.1	24.3	22.4	29.6		33.6		26.7		24.3
4,6-Man*p*		1.8	1.5	2.9	0.9	2.3		0.9		2.3		2.3
Total Man (M)		**29.0**	**25.8**	**30.6**	**25.1**	**33.7**		**33.7**		**33.0**		**30.0**
(A)		**(38.3)**	**(38.1)**	**(42.0)**	**(50.8)**	**(24.9)**		**(24.9)**		**(32.5)**		**(36.0)**
T-Gal*p*		13.1	13.1	13.1	12.7	10.7		9.2		11.1		12.6
6-Gal*p*		8.4	6.9	9.8	5.2	6.4		3.4		7.3		8.1
3-Gal*p*		26.4	31.4	25.9	35.6	24.6		30.3		25.0		24.4
3,6-Gal*p*		16.3	18.4	17.1	17.6	18.6		16.1		18.1		18.2
Total Gal (M)		**64.4**	**69.8**	**65.8**	**71.0**	**60.3**		**60.3**		**61.8**		**63.4**
(A)		**(51.4)**	**(51.8)**	**(49.8)**	**(42.5)**	**(61.9)**		**(61.9)**		**(57.0)**		**(54.6)**
T-Glc*p*		0.3	0.0	0.0	0.0	0.1		0.0		0.0		0.2
4-Glc*p*		0.2	1.3	0.9	2.0	1.1		1.1		1.0		3.2
Total Glc (M)		**0.5**	**1.3**	**0.9**	**2.0**	**1.2**		**1.2**		**1.0**		**3.4**
(A)		**(3.4)**	**(3.0)**	**(3.5)**	**(3.7)**	**(1.7)**		**(1.7)**		**(2.0)**		**(3.0)**
**AG**	***η***_**AG**_ (mg_AG_/g_SCG_)	69.8	68.8	93.2	95.7	64.8		56.4		95.4		58.7
	***η***_**AG**_ (%)^a^	69	71	66	72	63		62		64		64
	DP_AG_	4.8	5.2	4.8	5.5	5.4		6.3		5.4		4.8
	DB_AG_	0.26	0.27	0.27	0.25	0.32		0.28		0.31		0.30
**GM**	***η***_**GM**_ (mg_GM_/g_SCG_)	31.5	26.3	47.6	34.5	37.2		34.3		52.9		29.5
	***η***_**GM**_ (%)^b^	31	27	34	26	36		37		35		32
	DP_GM_	7.0	6.2	8.9	13.7	18.7		19.4		8.7		8.9
	DB_GM_	0.06	0.09	0.09	0.03	0.07		0.03		0.07		0.08
AG/GM			2.6	2.0	2.8	1.7		1.6		1.8		2.0

Samples A and B are the duplicate samples respectively obtained at reactor A and B in each microwave run. (M) Glycosidic-linkage composition of polysaccharides was determined as partially methylated alditol acetated by methylation analysis with GC-MS. (A) Sugar composition determined by derivatization to alditol acetates and analysis by GC-FID.

a [AG/(AG + GM)].

b [GM/(AG + GM)]. DP – Degree of polymerization. DB – Degree of Branching.

**Fig. 4:**
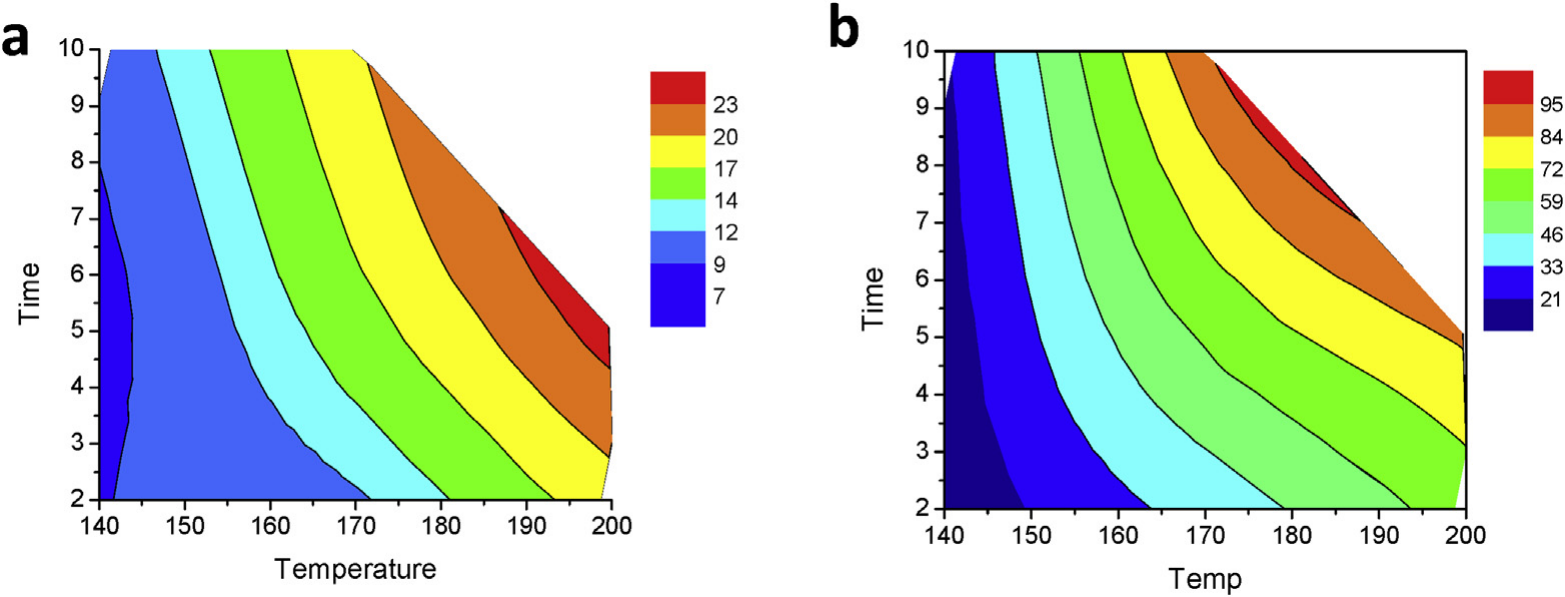
Contour plot representation of the interaction between the operating conditions, time (*t*) and temperature (*T*) for: a) total soluble solids mass yield [η_total soluble solids_, (%, w/w)]; and b) arabinogalactans content [η_AG_, (mg_AG_/g_SCG_)].

Section 1.3 shows a contour plot constructed using the data in Table 1, Table 2, Table 3, that can be used for optimization of extraction conditions, in particular maximization of mass yield (Fig. 4a) and arabinogalactans extraction (Fig. 4b).

The data for different microwave assisted extraction conditions in Table 1, Table 2, Table 3 were used for calculating ANOVA models in Ref. [1]. The results for the multiple comparisons with Bonferroni adjustment for these ANOVA models are presented in section 1.4 for total soluble solids mass yield (Table 4), total sugar yield (Table 5), arabinogalactans yield (Table 6), and galactomannans yield (Table 7).

### 1.1 GC-MS data of a mixture of arabinogalactans and galactomannans

Fig. 1 shows, as an example, a chromatogram of a mixture of galactomannans and arabinogalactans. Fig. 2 shows the corresponding mass spectra for each one of the major partially methylated alditol acetates identified in the chromatograms.

### 1.2 Map of dependence of polysaccharides glycosidic-linkage composition on the operating factors (treatment time and temperature) of microwave assisted treatment of spent coffee grounds

This section represents chromatograms (Fig. 3) and the respective data of sugars and glycosidic-linkage composition of samples obtained at different microwave assisted conditions (Table 1, Table 2, Table 3). These data were analyzed and discussed in Ref. [1]. Temperature effect is shown at 140 °C (Fig. 3a), 170 °C (Fig. 3b), and 200 °C (Fig. 3c). Effect of time of treatment is shown at 2 min (Fig. 3d), 5 min (Fig. 3b), and 10 min (Fig. 3e). Detailed information on glycosidic-linkage (M) and sugar composition (A) is in Table 1, Table 2, Table 3, for the samples at 140 °C (Table 1), 170 °C (Table 2), and 200 °C (Table 3).

### 1.3 Defining areas of similar applicability in accordance with maximum total mass yield (%, w/w) and maximum arabinogalactans’ yield

Contour plots are useful tool for visualization of the effects of two experimental factors on the parameter of interest when interaction between these two factors are present. Contour plots allow to define areas of similar applicability. E.g., maximum total soluble solids mass yield (%, w/w) was obtained using operating conditions 190–200 °C/4–7 min (Fig. 4a), while the maximum recovery of arabinogalactans was obtained under the operating conditions of 170–180 °C/7–9 min (Fig. 4b). Treatment conditions of 200 °C and 10 min were not considered in the data analysis as they were found to be too drastic, resulting in overpressure conditions in the microwave equipment (above 55 bar).

### 1.4 Pair-wise comparison of group means for experimental factors and their interactions using multiple comparison test with critical values from t distribution with Bonferroni adjustment

**Table 4:** Results of multiple comparison using Bonferroni test for total soluble solids mass yield.

Pairs	Lower confidence interval	Estimate of difference of means	Upper confidence interval	p-value
T(140) × T(170)	−8.32	−6.30	−4.29	0.000
T(140) × T(200)	−18.65	−15.73	−12.81	0.000
T(170) × T(200)	−12.34	−9.43	−6.51	0.000
t(2) × t(5)	−4.97	−2.57	−0.17	0.034
t(2) × t(10)	−9.13	−6.05	−2.97	0.000
t(5) × t(10)	−6.56	−3.48	−0.40	0.023
(No alkali) × (Alkali)	−0.19	1.40	2.99	0.082
T(140),t(2) × T(170),t(2)	−8.01	−2.88	2.26	1.000
T(140),t(2) × T(200),t(2)	−15.05	−9.92	−4.78	0.000
T(140),t(2) × T(140),t(5)	−4.38	0.76	5.89	1.000
T(140),t(2) × T(170),t(5)	−12.03	−6.90	−1.76	0.003
T(140),t(2) × T(200),t(5)	−20.68	−15.55	−10.42	0.000
T(140),t(2) × T(140),t(10)	−6.33	−1.20	3.93	1.000
T(140),t(2) × T(170),t(10)	−16.04	−10.91	−5.77	0.000
T(170), t(2) × T(200),t(2)	−12.17	−7.04	−1.91	0.002
T(170), t(2) × T(140),t(5)	−1.50	3.63	8.76	0.574
T(170), t(2) × T(170),t(5)	−9.15	−4.02	1.11	0.309
T(170), t(2) × T(200),t(5)	−17.81	−12.68	−7.54	0.000
T(170), t(2) × T(140),t(10)	−3.46	1.67	6.80	1.000
T(170), t(2) × T(170),t(10)	−13.16	−8.03	−2.90	0.000
T(200),t(2) × T(140),t(5)	5.54	10.67	15.80	0.000
T(200),t(2) × T(170),t(5)	−2.11	3.02	8.15	1.000
T(200),t(2) × T(2000),t(5)	−10.77	−5.64	−0.50	0.022
T(200),t(2) × T(140),t(10)	3.58	8.71	13.84	0.000
T(200),t(2) × T(170),t(10)	−6.12	−0.99	4.14	1.000
T(140),t(5) × T(170),t(5)	−12.78	−7.65	−2.52	0.001
T(140),t(5) × T(200),t(5)	−21.44	−16.31	−11.17	0.000
T(140),t(5) × T(140),t(10)	−7.09	−1.96	3.17	1.000
T(140),t(5) × T(170),t(10)	−16.79	−11.66	−6.53	0.000
T(170),t(5) × T(200),t(5)	−13.79	−8.66	−3.52	0.000
T(170),t(5) × T(140),t(10)	0.56	5.69	10.82	0.020
T(170),t(5) × T(170),t(10)	−9.14	−4.01	1.12	0.314
T(200),t(5) × T(140),t(10)	9.22	14.35	19.48	0.000
T(200),t(5) × T(170),t(10)	−0.49	4.65	9.78	0.112
T(140),t(10) × T(170),t(10)	−14.83	−9.70	−4.57	0.000
T(140),(No Alkali) × T(170),(No Alkali)	−8.78	−5.18	−1.57	0.002
T(140),(No Alkali) × T(2000),(No Alkali)	−22.09	−17.36	−12.63	0.000
T(140),(No Alkali) × T(140),(Alkali)	−2.17	1.43	5.03	1.000
T(140),(No Alkali) × T(170), (Alkali)	−9.63	−6.00	−2.38	0.000
T(140),(No Alkali) × T(200),(Alkali)	−17.35	−12.67	−7.99	0.000
T(170),(No Alkali) × T(200),(No Alkali)	−16.92	−12.19	−7.46	0.000
T(170),(No Alkali) × T(140),(Alkali)	2.98	6.61	10.23	0.000
T(170),(No Alkali) × T(170),(Alkali)	−4.43	−0.83	2.78	1.000
T(170),(No Alkali) × T(200),(Alkali)	−12.17	−7.49	−2.81	0.000
T(200),(No Alkali) × T(140),(Alkali)	14.11	18.79	23.47	0.000
T(200),(No Alkali) × T(170),(Alkali)	6.68	11.36	16.04	0.000
T(200),(No Alkali) × T(200),(Alkali)	0.15	4.70	9.24	0.039
T(140),(Alkali) × T(170),(Alkali)	−11.03	−7.43	−3.83	0.000
T(140),(Alkali) × T(200),(Alkali)	−18.83	−14.10	−9.37	0.000
T(170),(Alkali) × T(200),(Alkali)	−11.39	−6.66	−1.93	0.002
t(2),(No Alkali) × t(5),(No Alkali)	−6.73	−2.44	1.85	1.000
t(2),(No Alkali) × t(10),(No Alkali)	−10.74	−5.50	−0.26	0.034
t(2),(No Alkali) × t(2),(Alkali)	−2.52	1.77	6.06	1.000
t(2),(No Alkali) × t(5),(Alkali)	−5.23	−0.92	3.39	1.000
t(2),(No Alkali) × t(10),(Alkali)	−10.01	−4.83	0.35	0.085
t(5),(No Alkali) × t(10),(No Alkali)	−8.29	−3.06	2.18	1.000
t(5),(No Alkali) × t(2),(Alkali)	−0.10	4.21	8.52	0.060
t(5),(No Alkali) × t(5),(Alkali)	−2.77	1.52	5.81	1.000
t(5),(No Alkali) × t(10),(Alkali)	−7.57	−2.39	2.80	1.000
t(10),(No Alkali) × t(2),(Alkali)	2.08	7.27	12.45	0.002
t(10),(No Alkali) × t(5),(Alkali)	−0.61	4.58	9.76	0.123
t(10),(No Alkali) × t(10),(Alkali)	−4.72	0.67	6.05	1.000
t(2),(Alkali) × t(5),(Alkali)	−6.98	−2.69	1.60	0.763
t(2),(Alkali) × t(10),(Alkali)	−11.84	−6.60	−1.36	0.006
t(5),(Alkali) × t(10),(Alkali)	−9.15	−3.91	1.33	0.337

T – temperature (140 °C, 170 °C, 200 °C), t – time (2 min, 5 min, 10 min), Alkali – dilute alkali conditions or No Alkali – aqueous conditions.

**Table 5:** Results of multiple comparison using Bonferroni test for total sugar yield.

Pairs	Lower confidence interval	Estimate of difference of means	Upper confidence interval	p-value
T(140) × T(170)	−0.26	−0.19	−0.13	0.000
T(140) × T(200)	−0.21	−0.11	−0.02	0.020
T(170) × T(200)	−0.01	0.08	0.18	0.116
t(2) × t(5)	−0.13	−0.04	0.05	0.739
t(2) × t(10)	−0.28	−0.16	−0.04	0.006
t(5) × t(10)	−0.24	−0.12	0.00	0.053
(No alkali) × (Alkali)	−0.03	0.03	0.09	0.354
T(140),t(2) × T(170),t(2)	−0.37	−0.20	−0.04	0.008
T(140),t(2) × T(200),t(2)	−0.33	−0.16	0.01	0.067
T(140),t(2) × T(140),t(5)	−0.24	−0.07	0.10	1.000
T(140),t(2) × T(170),t(5)	−0.42	−0.25	−0.08	0.001
T(140),t(2) × T(200),t(5)	−0.33	−0.16	0.01	0.070
T(140),t(2) × T(140),t(10)	−0.27	−0.10	0.07	1.000
T(140),t(2) × T(170),t(10)	−0.46	−0.30	−0.13	0.000
T(170), t(2) × T(200),t(2)	−0.13	0.04	0.21	1.000
T(170), t(2) × T(140),t(5)	−0.04	0.13	0.30	0.296
T(170), t(2) × T(170),t(5)	−0.21	−0.04	0.13	1.000
T(170), t(2) × T(200),t(5)	−0.13	0.04	0.21	1.000
T(170), t(2) × T(140),t(10)	−0.06	0.11	0.27	1.000
T(170), t(2) × T(170),t(10)	−0.26	−0.09	0.08	1.000
T(200),t(2) × T(140),t(5)	−0.08	0.09	0.26	1.000
T(200),t(2) × T(170),t(5)	−0.25	−0.08	0.08	1.000
T(200),t(2) × T(2000),t(5)	−0.17	0.00	0.17	1.000
T(200),t(2) × T(140),t(10)	−0.10	0.06	0.23	1.000
T(200),t(2) × T(170),t(10)	−0.30	−0.13	0.03	0.287
T(140),t(5) × T(170),t(5)	−0.34	−0.18	−0.01	0.035
T(140),t(5) × T(200),t(5)	−0.26	−0.09	0.08	1.000
T(140),t(5) × T(140),t(10)	−0.20	−0.03	0.14	1.000
T(140),t(5) × T(170),t(10)	−0.39	−0.22	−0.06	0.003
T(170),t(5) × T(200),t(5)	−0.08	0.09	0.25	1.000
T(170),t(5) × T(140),t(10)	−0.02	0.15	0.32	0.138
T(170),t(5) × T(170),t(10)	−0.22	−0.05	0.12	1.000
T(200),t(5) × T(140),t(10)	−0.11	0.06	0.23	1.000
T(200),t(5) × T(170),t(10)	−0.30	−0.13	0.03	0.274
T(140),t(10) × T(170),t(10)	−0.37	−0.20	−0.03	0.012
T(140),(No Alkali) × T(170),(No Alkali)	−0.25	−0.14	−0.02	0.015
T(140),(No Alkali) × T(2000),(No Alkali)	−0.17	−0.01	0.14	1.000
T(140),(No Alkali) × T(140),(Alkali)	0.00	0.12	0.24	0.046
T(140),(No Alkali) × T(170), (Alkali)	−0.25	−0.13	−0.01	0.026
T(140),(No Alkali) × T(200),(Alkali)	−0.24	−0.09	0.07	1.000
T(170),(No Alkali) × T(200),(No Alkali)	−0.03	0.12	0.28	0.260
T(170),(No Alkali) × T(140),(Alkali)	0.14	0.26	0.38	0.000
T(170),(No Alkali) × T(170),(Alkali)	−0.11	0.01	0.13	1.000
T(170),(No Alkali) × T(200),(Alkali)	−0.11	0.05	0.20	1.000
T(200),(No Alkali) × T(140),(Alkali)	−0.02	0.13	0.29	0.130
T(200),(No Alkali) × T(170),(Alkali)	−0.27	−0.11	0.04	0.346
T(200),(No Alkali) × T(200),(Alkali)	−0.22	−0.07	0.08	1.000
T(140),(Alkali) × × T(170),(Alkali)	−0.37	−0.25	−0.13	0.000
T(140),(Alkali) × T(200),(Alkali)	−0.36	−0.21	−0.05	0.004
T(170),(Alkali) × T(200),(Alkali)	−0.11	0.04	0.20	1.000
t(2),(No Alkali) × t(5),(No Alkali)	−0.23	−0.07	0.10	1.000
t(2),(No Alkali) × t(10),(No Alkali)	−0.37	−0.17	0.03	0.180
t(2),(No Alkali) × t(2),(Alkali)	−0.16	0.01	0.17	1.000
t(2),(No Alkali) × t(5),(Alkali)	−0.18	−0.01	0.16	1.000
t(2),(No Alkali) × t(10),(Alkali)	−0.34	−0.15	0.05	0.368
t(5),(No Alkali) × t(10),(No Alkali)	−0.30	−0.10	0.10	1.000
t(5),(No Alkali) × t(2),(Alkali)	−0.09	0.07	0.24	1.000
t(5),(No Alkali) × t(5),(Alkali)	−0.11	0.06	0.22	1.000
t(5),(No Alkali) × t(10),(Alkali)	−0.28	−0.08	0.12	1.000
t(10),(No Alkali) × t(2),(Alkali)	−0.03	0.17	0.37	0.138
t(10),(No Alkali) × t(5),(Alkali)	−0.04	0.16	0.36	0.246
t(10),(No Alkali) × t(10),(Alkali)	−0.19	0.02	0.23	1.000
t(2),(Alkali) × t(5),(Alkali)	−0.18	−0.02	0.15	1.000
t(2),(Alkali) × t(10),(Alkali)	−0.35	−0.15	0.05	0.321
t(5),(Alkali) × t(10),(Alkali)	−0.34	−0.14	0.07	0.552

T – temperature (140 °C, 170 °C, 200 °C), t – time (2 min, 5 min, 10 min), Alkali – dilute alkali conditions or No Alkali – aqueous conditions.

**Table 6:** Results of multiple comparison using Bonferroni test for arabinogalactans yield.

Pairs	Lower confidence interval	Estimate of difference of means	Upper confidence interval	p-value
T(140) × T(170)	−57.6	−46.8	−35.9	0.000
T(140) × T(200)	−88.5	−72.7	−57.0	0.000
T(170) × T(200)	−41.7	−26.0	−10.3	0.001
t(2) × t(5)	−27.8	−15.0	−2.3	0.017
t(2) × t(10)	−66.6	−50.3	−34.0	0.000
t(5) × t(10)	−51.6	−35.3	−18.9	0.000
(No alkali) × (Alkali)	−7.3	1.4	10.2	0.738
T(140),t(2) × T(170),t(2)	−56.5	−27.8	0.8	0.064
T(140),t(2) × T(200),t(2)	−83.4	−54.8	−26.1	0.000
T(140),t(2) × T(140),t(5)	−32.8	−4.2	24.5	1.000
T(140),t(2) × T(170),t(5)	−79.8	−51.1	−22.5	0.000
T(140),t(2) × T(200),t(5)	−104.2	−75.6	−47.0	0.000
T(140),t(2) × T(140),t(10)	−37.4	−8.8	19.8	1.000
T(140),t(2) × T(170),t(10)	−112.4	−83.8	−55.1	0.000
T(170), t(2) × T(200),t(2)	−55.6	−26.9	1.7	0.084
T(170), t(2) × T(140),t(5)	−5.0	23.7	52.3	0.216
T(170), t(2) × T(170),t(5)	−51.9	−23.3	5.4	0.244
T(170), t(2) × T(200),t(5)	−76.4	−47.8	−19.1	0.000
T(170), t(2) × T(140),t(10)	−9.6	19.0	47.7	0.806
T(170), t(2) × T(170),t(10)	−84.6	−55.9	−27.3	0.000
T(200),t(2) × T(140),t(5)	21.9	50.6	79.2	0.000
T(200),t(2) × T(170),t(5)	−25.0	3.6	32.3	1.000
T(200),t(2) × T(2000),t(5)	−49.5	−20.8	7.8	0.488
T(200),t(2) × T(140),t(10)	17.3	45.9	74.6	0.000
T(200),t(2) × T(170),t(10)	−57.7	−29.0	−0.4	0.045
T(140),t(5) × T(170),t(5)	−75.6	−47.0	−18.3	0.000
T(140),t(5) × T(200),t(5)	−100.1	−71.4	−42.8	0.000
T(140),t(5) × T(140),t(10)	−33.3	−4.6	24.0	1.000
T(140),t(5) × T(170),t(10)	−108.3	−79.6	−51.0	0.000
T(170),t(5) × T(200),t(5)	−53.1	−24.5	4.2	0.171
T(170),t(5) × T(140),t(10)	13.7	42.3	71.0	0.001
T(170),t(5) × T(170),t(10)	−61.3	−32.7	−4.0	0.015
T(200),t(5) × T(140),t(10)	38.2	66.8	95.4	0.000
T(200),t(5) × T(170),t(10)	−36.8	−8.2	20.5	1.000
T(140),t(10) × T(170),t(10)	−103.6	−75.0	−46.3	0.000
T(140),(No Alkali) × T(170),(No Alkali)	−60.6	−41.1	−21.7	0.000
T(140),(No Alkali) × T(2000),(No Alkali)	−104.3	−78.8	−53.3	0.000
T(140),(No Alkali) × T(140),(Alkali)	−16.8	2.6	22.0	1.000
T(140),(No Alkali) × T(170), (Alkali)	−69.3	−49.7	−30.2	0.000
T(140),(No Alkali) × T(200),(Alkali)	−89.3	−64.1	−38.8	0.000
T(170),(No Alkali) × T(200),(No Alkali)	−63.1	−37.6	−12.1	0.001
T(170),(No Alkali) × T(140),(Alkali)	24.2	43.8	63.3	0.000
T(170),(No Alkali) × T(170),(Alkali)	−28.0	−8.6	10.8	1.000
T(170),(No Alkali) × T(200),(Alkali)	−48.2	−22.9	2.3	0.101
T(200),(No Alkali) × T(140),(Alkali)	56.2	81.4	106.6	0.000
T(200),(No Alkali) × T(170),(Alkali)	3.8	29.0	54.3	0.015
T(200),(No Alkali) × T(200),(Alkali)	−9.8	14.7	39.2	0.914
T(140),(Alkali) × T(170),(Alkali)	−71.8	−52.4	−32.9	0.000
T(140),(Alkali) × T(200),(Alkali)	−92.2	−66.7	−41.2	0.000
T(170),(Alkali) × T(200),(Alkali)	−39.8	−14.3	11.2	1.000
t(2),(No Alkali) × t(5),(No Alkali)	−37.6	−14.9	7.9	0.643
t(2),(No Alkali) × t(10),(No Alkali)	−80.3	−52.5	−24.8	0.000
t(2),(No Alkali) × t(2),(Alkali)	−22.3	0.5	23.2	1.000
t(2),(No Alkali) × t(5),(Alkali)	−37.6	−14.8	8.1	0.674
t(2),(No Alkali) × t(10),(Alkali)	−75.1	−47.6	−20.1	0.000
t(5),(No Alkali) × t(10),(No Alkali)	−65.4	−37.7	−9.9	0.003
t(5),(No Alkali) × t(2),(Alkali)	−7.5	15.3	38.2	0.572
t(5),(No Alkali) × t(5),(Alkali)	−22.6	0.1	22.8	1.000
t(5),(No Alkali) × t(10),(Alkali)	−60.2	−32.8	−5.3	0.011
t(10),(No Alkali) × t(2),(Alkali)	25.5	53.0	80.5	0.000
t(10),(No Alkali) × t(5),(Alkali)	10.3	37.8	65.2	0.003
t(10),(No Alkali) × t(10),(Alkali)	−23.6	4.9	33.4	1.000
t(2),(Alkali) × t(5),(Alkali)	−38.0	−15.2	7.5	0.572
t(2),(Alkali) × t(10),(Alkali)	−75.9	−48.1	−20.3	0.000
t(5),(Alkali) × t(10),(Alkali)	−60.6	−32.8	−5.1	0.012

T – temperature (140 °C, 170 °C, 200 °C), t – time (2 min, 5 min, 10 min), Alkali – dilute alkali conditions or No Alkali – aqueous conditions.

**Table 7:** Results of multiple comparison using Bonferroni test for galactomannans yield.

Pairs	Lower confidence interval	Estimate of difference of means	Upper confidence interval	p-value
T(140) × T(170)	−16.8	−9.4	−1.9	0.011
T(140) × T(200)	−29.4	−18.7	−8.0	0.001
T(170) × T(200)	−20.1	−9.3	1.4	0.104
t(2) × t(5)	−11.3	−4.2	3.0	0.433
t(2) × t(10)	−16.9	−7.8	1.3	0.113
t(5) × t(10)	−12.7	−3.6	5.5	0.942
(No alkali) × (Alkali)	1.4	6.2	11.1	0.013
T(140),t(2) × T(170),t(2)	−24.6	−6.0	12.6	1.000
T(140),t(2) × T(200),t(2)	−29.0	−10.4	8.3	1.000
T(140),t(2) × T(140),t(5)	−17.8	0.8	19.4	1.000
T(140),t(2) × T(170),t(5)	−30.9	−12.2	6.4	0.852
T(140),t(2) × T(200),t(5)	−37.8	−19.2	−0.6	0.039
T(140),t(2) × T(140),t(10)	−22.8	−4.2	14.4	1.000
T(140),t(2) × T(170),t(10)	−32.4	−13.8	4.8	0.441
T(170), t(2) × T(200),t(2)	−22.9	−4.3	14.3	1.000
T(170), t(2) × T(140),t(5)	−11.8	6.8	25.4	1.000
T(170), t(2) × T(170),t(5)	−24.8	−6.2	12.4	1.000
T(170), t(2) × T(200),t(5)	−31.8	−13.1	5.5	0.580
T(170), t(2) × T(140),t(10)	−16.8	1.8	20.4	1.000
T(170), t(2) × T(170),t(10)	−26.4	−7.8	10.9	1.000
T(200),t(2) × T(140),t(5)	−7.5	11.1	29.8	1.000
T(200),t(2) × T(170),t(5)	−20.5	−1.9	16.7	1.000
T(200),t(2) × T(2000),t(5)	−27.4	−8.8	9.8	1.000
T(200),t(2) × T(140),t(10)	−12.5	6.1	24.7	1.000
T(200),t(2) × T(170),t(10)	−22.0	−3.4	15.2	1.000
T(140),t(5) × T(170),t(5)	−31.6	−13.0	5.6	0.611
T(140),t(5) × T(200),t(5)	−38.6	−20.0	−1.3	0.027
T(140),t(5) × T(140),t(10)	−23.6	−5.0	13.6	1.000
T(140),t(5) × T(170),t(10)	−33.2	−14.6	4.1	0.313
T(170),t(5) × T(200),t(5)	−25.5	−6.9	11.7	1.000
T(170),t(5) × T(140),t(10)	−10.6	8.0	26.6	1.000
T(170),t(5) × T(170),t(10)	−20.2	−1.5	17.1	1.000
T(200),t(5) × T(140),t(10)	−3.7	14.9	33.6	0.264
T(200),t(5) × T(170),t(10)	−13.2	5.4	24.0	1.000
T(140),t(10) × T(170),t(10)	−28.2	−9.6	9.1	1.000
T(140),(No Alkali) × T(170),(No Alkali)	−17.7	−4.5	8.8	1.000
T(140),(No Alkali) × T(2000),(No Alkali)	−26.6	−9.2	8.2	1.000
T(140),(No Alkali) × T(140),(Alkali)	1.4	14.7	27.9	0.022
T(140),(No Alkali) × T(170), (Alkali)	−12.9	0.4	13.8	1.000
T(140),(No Alkali) × T(200),(Alkali)	−30.8	−13.6	3.7	0.251
T(170),(No Alkali) × T(200),(No Alkali)	−22.1	−4.7	12.7	1.000
T(170),(No Alkali) × T(140),(Alkali)	5.8	19.1	32.5	0.002
T(170),(No Alkali) × T(170),(Alkali)	−8.4	4.9	18.2	1.000
T(170),(No Alkali) × T(200),(Alkali)	−26.3	−9.1	8.1	1.000
T(200),(No Alkali) × T(140),(Alkali)	6.6	23.8	41.1	0.002
T(200),(No Alkali) × T(170),(Alkali)	−7.6	9.6	26.8	1.000
T(200),(No Alkali) × T(200),(Alkali)	−21.1	−4.4	12.4	1.000
T(140),(Alkali) × T(170),(Alkali)	−27.5	−14.2	−1.0	0.028
T(140),(Alkali) × T(200),(Alkali)	−45.6	−28.2	−10.8	0.000
T(170),(Alkali) × T(200),(Alkali)	−31.4	−14.0	3.4	0.224
t(2),(No Alkali) × t(5),(No Alkali)	−19.1	−6.5	6.2	1.000
t(2),(No Alkali) × t(10),(No Alkali)	−21.0	−5.5	10.0	1.000
t(2),(No Alkali) × t(2),(Alkali)	−7.0	5.6	18.3	1.000
t(2),(No Alkali) × t(5),(Alkali)	−9.0	3.8	16.5	1.000
t(2),(No Alkali) × t(10),(Alkali)	−19.7	−4.4	10.9	1.000
t(5),(No Alkali) × t(10),(No Alkali)	−14.6	0.9	16.4	1.000
t(5),(No Alkali) × t(2),(Alkali)	−0.7	12.1	24.8	0.075
t(5),(No Alkali) × t(5),(Alkali)	−2.4	10.2	22.9	0.214
t(5),(No Alkali) × t(10),(Alkali)	−13.3	2.1	17.4	1.000
t(10),(No Alkali) × t(2),(Alkali)	−4.2	11.2	26.5	0.382
t(10),(No Alkali) × t(5),(Alkali)	−6.0	9.3	24.6	0.870
t(10),(No Alkali) × t(10),(Alkali)	−14.8	1.1	17.1	1.000
t(2),(Alkali) × t(5),(Alkali)	−14.5	−1.8	10.8	1.000
t(2),(Alkali) × t(10),(Alkali)	−25.5	−10.0	5.5	0.669
t(5),(Alkali) × t(10),(Alkali)	−23.7	−8.2	7.3	1.000

T – temperature (140 °C, 170 °C, 200 °C), t – time (2 min, 5 min, 10 min), Alkali – dilute alkali conditions or No Alkali – aqueous conditions.

## 2 Experimental design, materials and methods

The details on the experimental design for microwave assisted treatments, methods for sugar analysis, and glycosidic-linkage analysis measurements are described in Ref. [1] and detailed in Refs. [3], [4], [5].

### 2.1 Microwave irradiation

A MicroSYNTH Labstation (Milestone srl., Bergamo, Italy) equipment with a maximum output delivery power of 1000 W was used for the microwave experiments using two high pressure reactors of 100 mL capacity each. The MicroSYNTH Labstation is a multimode microwave oven in which the real-time temperature inside the reactor is monitored with a thermometer. Heating temperature is controlled precisely with a PID (Proportional, Integral, Derivative) algorithm by changing the power of microwave irradiation. The suspension in the reactor is continuously stirred with a magnetic bar that minimizes the heterogeneous microwave heating. The reactor is made of polytetrafluoroethylene (PTFE) containing <1% perfluoropropyl vinyl ether (PPVE) modifier that can endure temperatures up to 250 °C and pressures up to 55 bar. Microwave energy is transmitted through the reactor and directly heats the compounds inside.

Each experiment was conducted in two similar reactors standing opposite to each other. Suspensions containing the proportion of 1:10 of spent coffee grounds (SCG) (dry weight, g) and water (mL) or in case of alkali dilute conditions (0.1 M KOH) were prepared in a total volume of approximately 70 mL. Microwave power was adjusted to attain 140, 170, and 200 °C in 3 min, and maintain the temperature for 2, 5, or 10 min. Due to security measures the equipment was programmed to stop irradiating whenever the temperature overcame the one displayed and/or when pressure achieved 40 bar. The reactors were cooled down to room temperature. All samples were centrifuged at 15 000 rpm, for 20 min, at 4 °C and the supernatant solution was filtered using MN GF-3 glass fibre filter, frozen, freeze-dried, and stored under an anhydrous atmosphere.

### 2.2 Sugar analysis

The total sugars content was determined by the sum of the amount of the individual sugars, taking into account that the hydrolysis of a glycosidic linkage results in an addition of a water molecule into the sugar structure. The polysaccharides were treated with 12 M H_2_SO_4_ for 3 h (room temperature) with occasional stirring followed by hydrolysis with 2 M H_2_SO_4_ at 120 °C during 1 h. Monosaccharides were reduced with NaBH_4_ (15%, NH_3_ 3 M) at 30 °C during 1 h and acetylated with acetic anhydride (3 mL) in the presence of 1-methylimidazole (450 μL) at 30 °C during 30 min. Alditol acetate derivatives were separated with dichloromethane and analyzed by GC with a FID detector (Perkin Elmer – Clarus 400) and equipped with a 30 m column DB-225 (i.d. 0.25 mm, film thickness of 0.15 μm) (J&W Scientific, Folsom, CA, USA). The oven temperature program used was: initial temperature 200 °C, a rise in temperature at a rate of 40 °C/min until 220 °C, standing for 7 min, followed by a rate of 20 °C/min until 230 °C and maintaining this temperature 1 min. The injector and detector temperatures were, respectively, 220 and 230 °C. The flow rate of the carrier gas (H_2_) was set at 1.7 mL/min [3]. The hydrolysis of all samples was performed in duplicate. In cases where the major sugars had higher than 5% variability a third analysis was performed.

### 2.3 Glycosidic-linkage analysis

Glycosidic-linkage composition of polysaccharides was determined by methylation analysis [3], [6]. The samples (1–2 mg) were dissolved in 1 mL of anhydrous dimethylsulfoxide (DMSO), then powdered NaOH (40 mg) were added under an argon atmosphere. The samples were methylated with CH_3_I (80 μL) during 20 min with stirring, following by a second addition of 80 μL CH_3_I and stirring for another 20 min. CHCl_3_/MeOH (1:1, v/v, 3 mL) was added, and the solution was dialyzed (membrane with a pore diameter of 12–14 kDa) against 3 lots of 50% EtOH. The dialysate was evaporated to dryness and the material was remethylated using the same procedure. The remethylated material was hydrolyzed with 2 M TFA (1 mL) at 120 °C for 1 h, and then reduced and acetylated as previously described for neutral sugar analysis (using NaBD_4_ instead of NaBH_4_).

### 2.4 GC-MS chromatographic conditions

The partially methylated alditol acetates (PMAA) were separated and analyzed by gas chromatography–mass spectrometry (GC–MS) (Agilent Technologies 6890 N Network). The GC was equipped with a DB-1 (J&W Scientific, Folsom, CA, USA) capillary column (30 m length, 0.25 mm of internal diameter, and 0.10 μm of film thickness). The samples were injected in pulsed splitless mode (time of splitless 5 min), with the injector operating at 220 °C, and using the following temperature program: 50 °C with a linear increase of 8 °C/min up to 140 °C, and standing for 5 min at this temperature, followed by a linear increase of 0.5 °C/min up to 150 °C, followed by a linear increase of 40 °C/min up to 250 °C, with further 1 min at 250 °C. The helium carrier gas had a flow rate of 1.7 mL/min, linear average velocity 48 cm s^−1^ and a column head pressure of 14.4 psi. The transfer line temperature of 300 °C. The GC was connected to an Agilent 5973 mass quadrupole selective detector operating with an electron impact mode at 70 eV and scanning the range *m*/*z* 50–550 with 3.25 scans min^−1^ in a full scan mode acquisition.

The equations used for calculations of the Degree of Polymerization (DB) and Degree of Branching (DB) were based on cited ref. [7], [8], which are also described in Ref. [1].

All calculation were made in Matlab 9.5 (R2018b).
