# Supplementary material for: Data on yields, sugars and glycosidic-linkage analyses of coffee arabinogalactan and galactomannan mixtures and optimization of their microwave assisted extraction from spent coffee grounds
Source: Data Brief. 2019 Apr 19;24:103931. doi: 10.1016/j.dib.2019.103931 (PMC6514364; doi:10.1016/j.dib.2019.103931)
Supplement: Supplementary file 1 — Multimedia Component 1 [file mmc1.docx]

**AUTHOR DECLARATION**

Aveiro, 22^nd^ March 2019

I confirm that there are no known conflicts of interest associated with this publication.

I confirm that the manuscript has been read and approved by all named authors and that there are no other persons who satisfied the criteria for authorship but are not listed. I further confirm that the order of authors listed in the manuscript has been approved by all of us.

Yours sincerely,

Cláudia P. Passos
